# Mapping the human membrane proteome: a majority of the human membrane proteins can be classified according to function and evolutionary origin

**DOI:** 10.1186/1741-7007-7-50

**Published:** 2009-08-13

**Authors:** Markus Sällman Almén, Karl JV Nordström, Robert Fredriksson, Helgi B Schiöth

**Affiliations:** 1 Department of Neuroscience, Functional Pharmacology, Uppsala University, Uppsala, Sweden

## Abstract

**Background:**

Membrane proteins form key nodes in mediating the cell's interaction with the surroundings, which is one of the main reasons why the majority of drug targets are membrane proteins.

**Results:**

Here we mined the human proteome and identified the membrane proteome subset using three prediction tools for alpha-helices: Phobius, TMHMM, and SOSUI. This dataset was reduced to a non-redundant set by aligning it to the human genome and then clustered with our own interactive implementation of the ISODATA algorithm. The genes were classified and each protein group was manually curated, virtually evaluating each sequence of the clusters, applying systematic comparisons with a range of databases and other resources. We identified 6,718 human membrane proteins and classified the majority of them into 234 families of which 151 belong to the three major functional groups: receptors (63 groups, 1,352 members), transporters (89 groups, 817 members) or enzymes (7 groups, 533 members). Also, 74 miscellaneous groups with 697 members were determined. Interestingly, we find that 41% of the membrane proteins are singlets with no apparent affiliation or identity to any human protein family. Our results identify major differences between the human membrane proteome and the ones in unicellular organisms and we also show a strong bias towards certain membrane topologies for different functional classes: 77% of all transporters have more than six helices while 60% of proteins with an enzymatic function and 88% receptors, that are not GPCRs, have only one single membrane spanning α-helix. Further, we have identified and characterized new gene families and novel members of existing families.

**Conclusion:**

Here we present the most detailed roadmap of gene numbers and families to our knowledge, which is an important step towards an overall classification of the entire human proteome. We estimate that 27% of the total human proteome are alpha-helical transmembrane proteins and provide an extended classification together with in-depth investigations of the membrane proteome's functional, structural, and evolutionary features.

## Background

Integral membrane proteins play a key role in detecting and conveying outside signals into cells, allowing them to interact and respond to their environment in a specific manner. They form principal nodes in hormonal and neuronal signaling and attract large interest in therapeutic interventions as the majority of drug targets are associated to the cell's membrane. Although the human genome has been public for several years, the exact number and identity of all protein coding genes have been hard to determine [1]. One of the most referenced papers regarding the percentage of membrane proteins in proteomes is from 2001 where the membrane topology prediction method TMHMM was applied on a number of proteomes from different species to estimate the membrane protein content, for example, *Caenorhabditis elegans *(31%), *Escherichia coli *(21%) and *Drosophila melanogaster *(20%) [2]. However, the human or any other vertebrate's proteome was not included in this study. The original human genome sequence project estimated 20% of the total gene count of 31,778 genes to code for membrane proteins [3]. More recently, four commonly used membrane topology prediction methods were applied to the human proteome [4]. Based on the range of predictions by the different methods 15 to 39% of the human proteome was dedicated to be membrane proteins, clearly illustrating how difficult it is to estimate the number with automatic approaches. The membrane proteomes of *E. coli *and *Saccharomyces cerevisiae *have previously been described in a fairly comprehensive manner [5,6]. Recent overviews of membrane bound proteins discuss important membrane protein groups such as the G-protein coupled receptors (GPCR), Aquaporins, Ion channels, ATPases, their structure and topology [7,8]. While several individual protein and gene families have been relatively well described, for example, the GPCRs [9] and Voltage-gated ion channels [10], there is a considerable number of genes that have remained unexplored.

We report the first detailed roadmap of the gene repertoire of human membrane bound proteins. We used 69,731 protein sequences from the International Protein Index (IPI) dataset, representing the total human proteome, to create an informative classification for the majority of the non-redundant transmembrane (TM) proteins. IPI is a top level domain, aiming to provide a union of the primary resources for proteins, as such it can be considered to contain all known protein sequences to current knowledge [11]. The analysis was performed in a two-step classification procedure, involving automatic prediction and classification *in silico*, combined with manual curation for each of the protein groups, virtually sequence for sequence applying systematic comparisons with a range of databases and other resources. We find that a large proportion of the membrane proteins can be assigned a function either as receptors, transporters, or enzymes and that a majority of all membrane proteins can be assigned to a family of evolutionary related proteins while 41% of the membrane proteins are found as single genes without close relatives. Furthermore, we describe and classify new protein groups and novel members of existing families, such as the putative solute carrier family AMAC and a novel putative calcium-channel gamma subunit.

## Results

**Figure 1:**
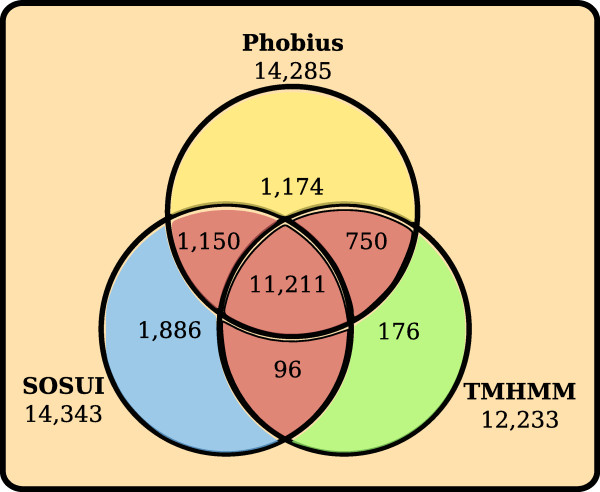
**Schematic diagram of the transmembrane protein prediction results**. Three different transmembrane prediction applications were applied on the International Protein Index (IPI) Human v.3.39 dataset of 69,731 protein sequences. A consensus approach, where only the proteins predicted as transmembrane by two applications (red color in diagram) were considered, resulted in 13,208 predicted transmembrane proteins.

**Figure 2:**
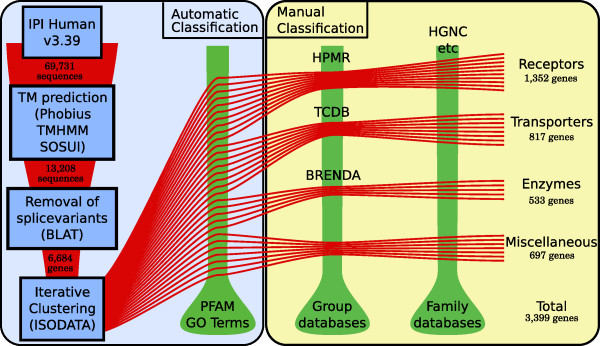
**Schematic overview of the classification process of all human membrane proteins**. The classification process had two general steps: an automatic and a manual or semi-manual. The automatic step can be divided into four parts, represented by blue boxes. First a dataset representing the human proteome was downloaded from the International Protein Index Transmembrane proteins were predicted from the proteome by using three different TM helix prediction softwares: Phobius, SOSUI, and TMHMM. Proteins predicted to contain at least one TM helix by two of the softwares were assigned for further analysis. Splice variants were removed using BLAT to align all protein sequences to the human genome. The longest protein sequence for each genomic location, defined as a gene, was selected and clustered using a local implementation of the ISODATA algorithm. Pfam and GO terms, describing molecular function, were downloaded from IPI and used to provide an initial view of the created clusters' function and family affiliation. This information was used to divide them into three functional classes (receptors, enzymes, and transporters) and one miscellaneous class. In the manual classification step the clusters were compared with group databases, specialized in the three functional groups, and to family databases that provide information about protein families and their members. These resources are shown by the green bars in the figure. By combining the results from the clustering with members found in databases a final result could be compiled for the different protein families and groups.

We created a dataset of 13,208 human membrane proteins based on consensus predictions of α-helices with three applications, Phobius [12], TMHMM [2], and SOSUI [13], in all 69,731 sequences in the human proteome dataset provided by IPI (v3.39). The predictions of the individual applications and the consensus approach are shown in Figure 1. The dataset was reduced to a non-redundant set of 6,684 protein sequences by aligning all predicted membrane proteins to the human genome with BLAT [14] and removing all but the longest representative protein for each genomic location. The non-redundant dataset was categorized into groups using an automatic approach where all protein sequences were compared with each other and clustered according to similarity with our own implementation of the ISODATA algorithm combined with manual curation where data from literature and public databases were considered. This extended the final membrane protein dataset to cover 6,718 proteins, with 3,399 proteins of the final membrane dataset being categorized into one of 234 protein families or groups and assigned a functional class (see Figure 2 and *Materials and Methods *for more details). To determine the quality of our classification we used the sequence comparisons from the clustering to find the median identity for the best hit among the classified and the unclassified proteins, respectively. The classified proteins have a significant median identity greater than 35% (*P *< 0.001) and the unclassified have a median identity less than 13% (*P *< 0.001) using a non-parametric Wilcoxon signed rank test.

Recently, Clamp and colleagues provided a new updated set of the human gene catalog [1]. This dataset contains a total of 23,789 genes from the Ensembl catalog of which 19,523 are classified as valid protein-coding. We used this dataset to classify our membrane proteins as true protein coding or non-coding. Our analysis shows that 956 proteins of our membrane protein dataset were not present in Clamp's dataset. They are considered to be invalid protein coding genes together with the 402 proteins which represent confirmed invalid genes. When the invalid proteins are excluded, 5,359 proteins remain of our membrane protein dataset. This exclusion confirms the strength and quality of our classification as 1,106, or 81%, of the invalid genes constitute 33% of the unclassified proteins. Thus, when making this more stringent estimation of protein numbers, we have classified 3,145 (59%) of the valid membrane proteins.

### Receptors

**Figure 3:**
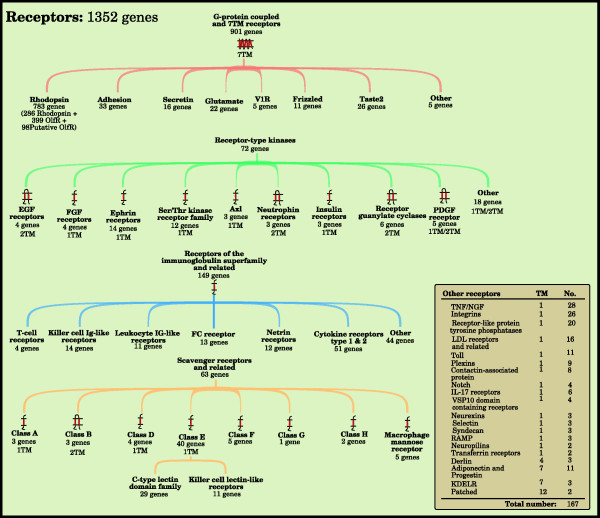
**The human receptors**. The figure shows the major families of membrane proteins that are classified to primarily function as receptors, proteins that trigger a cellular response upon binding of specific ligands. The tree structures give a comprehensive view of subfamilies in the largest families. The number of genes and function for each family have been determined by combining results from clustering, using the ISODATA algorithm, with data from the literature and public databases. Primarily the Human Plasma Membrane Receptome database and HGNC have been used. Consensus TM helix numbers have been set through evaluation of data from external resources among the literature and databases together with prediction results from Phobius, SOSUI, and TMHMM. The Other table contains receptor families that do not fit into any of the larger receptor families.

A receptor is a protein that mediates a cellular response upon binding of a ligand. We identified 1,352 proteins as receptors and divided them into 63 groups (Figure 3). Most of these families can be placed in one of four superfamilies; *G protein-coupled receptors *(901 proteins), *Receptor type tyrosine kinases *(72 proteins), *Receptors of the immunoglobulin superfamily and related *(149 proteins) and *Scavenger receptors and related *(63 proteins). The remaining 167 receptors, in 20 groups, are annotated as *Other receptors*.

### Transporters

**Figure 4:**
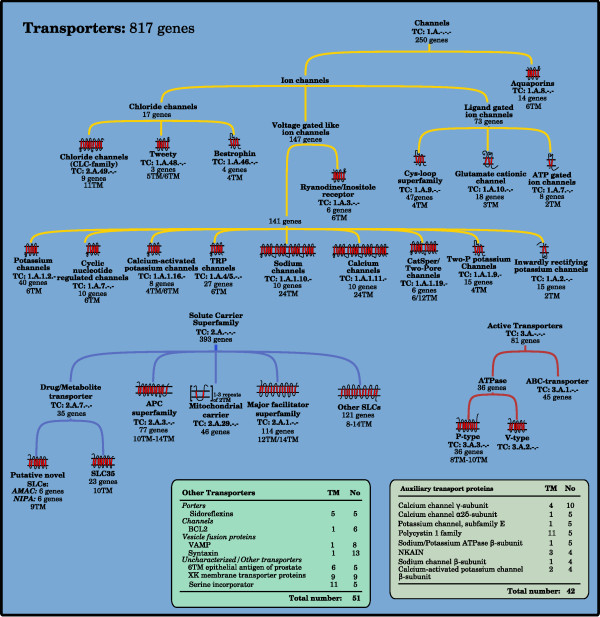
**The human transporters**. The figure shows the major families of membrane proteins that primarily function as transporters, proteins that facilitate the movement of substrates across a membrane. The tree structures represent three major classes of transporters that differ in energy dependence and utilization during transport. The number of genes and function for each family have been determined by combining results from clustering, using the ISODATA algorithm, with data from the literature and public databases. Primarily TCDB and HGNC were used. Consensus TM helix numbers have been set through evaluation of data from external resources among the literature and databases together with prediction results from Phobius, SOSUI, and TMHMM. The Other table contains transporter families that do not fit into any of the larger families. The table of Auxiliary transport proteins contains families that are not essential for transport, but modulate other transport proteins through different interactions.

Transporters perform the movement of a substrate across membranes by utilizing electrochemical gradients or energy from chemical reactions. We identified 817 transporters and placed them in 89 groups (Figure 4). The groups have been primarily arranged into three major functional classes: *Channels *(247 proteins), *Solute carriers *(393 proteins), and *Active transporters *(81 proteins). The remaining eight groups are annotated as *Other transporters *(51 proteins). Here we also include 42 auxiliary transport proteins in 9 groups that modulate the activity of other transporters rather than performing the transport themselves.

### Enzymes

**Figure 5:**
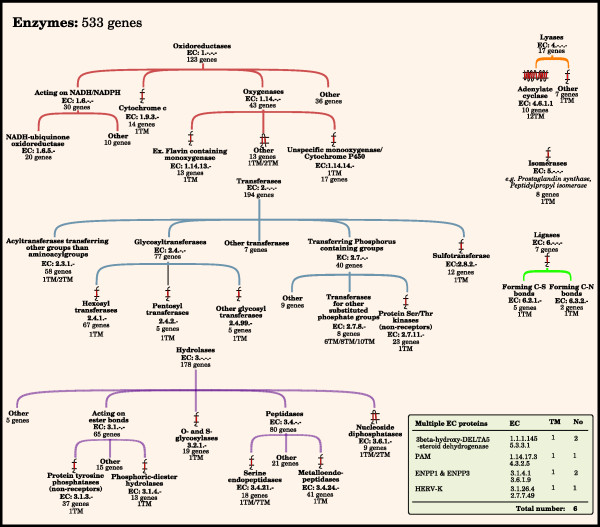
**The human enzymes**. The figure shows the major families of membrane proteins that primarily function as enzymes, proteins that catalyze a chemical reaction. The enzymes were divided into six major classes, depending of the character of the chemical reaction they are involved in. This was performed according to the EC system. Each of the six classes is represented by a tree structure, showing some of the hierarchical order of subclasses within each class. The number of genes for each class and subclass, and function, have been determined by combining results from clustering, using the ISODATA algorithm, with data from the literature and public databases. Primarily the BRENDA database was used. Consensus TM helix numbers have been set through evaluation of data from external resources among the literature and databases together with prediction results from Phobius, SOSUI, and TMHMM. The box contains proteins that belong to more than one class.

Enzymes are proteins with the ability to catalyze a chemical reaction. We have identified 533 enzymes (Figure 5). They have been classified based on the EC system, which classifies enzymes performing a similar type of reaction into six major classes: *Oxidoreductases *(123 proteins), *Transferases *(194 proteins), *Hydrolases *(178 proteins), *Lyases *(17 proteins), *Isomerases *(8 proteins) and *Ligases *(7 proteins). An additional six enzymes belong to multiple classes.

### Miscellaneous

The 74 protein families that did not fit into any of the 3 major functional classes were gathered in a class called Miscellaneous. This class contains 697 proteins and is further divided into four subclasses: *Ligands *(57 proteins), *Other *(272 proteins), *Structural/Adhesion proteins *(187 proteins) and *Proteins of unknown function *(181 proteins).

### Membrane topology

**Figure 6:**
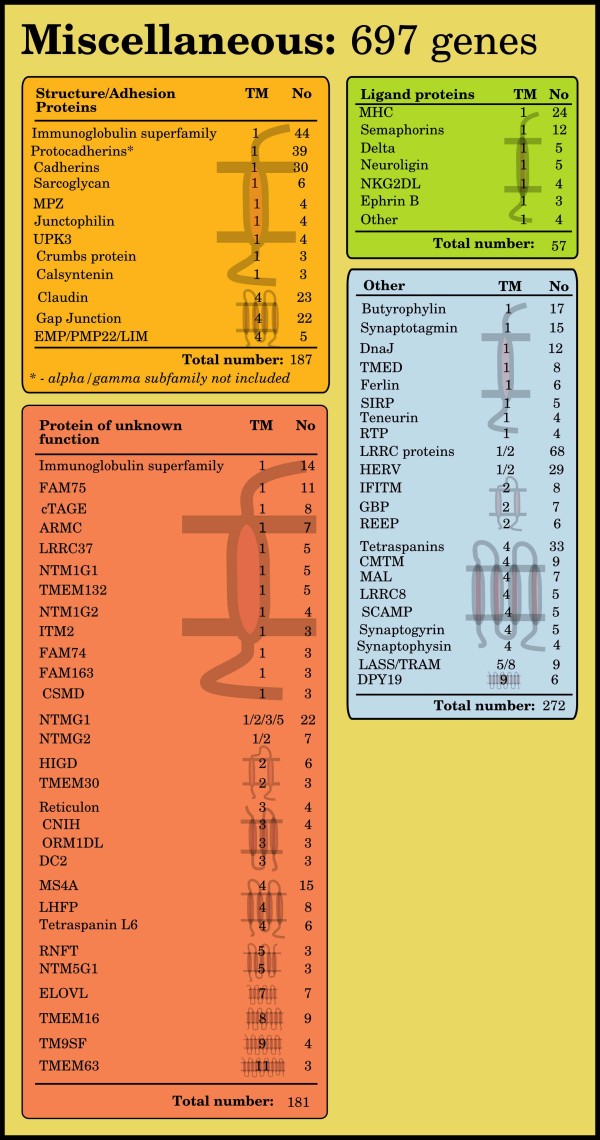
**Miscellaneous human protein groups**. The five boxes represent functional classes of proteins that do not fit within the definition of receptors, enzymes, or transporters. Structure/Adhesion proteins are those that build up structure between or within cells or mediate adhesion between the cell and the surroundings. Ligand proteins are groups that mainly function as ligands, structures that bind to receptors. Vesicle membrane proteins are proteins found in the membrane of cellular vesicles. The large Other group are protein groups of various functions that do not fit together with the other groups. Proteins of 'unknown function' show groups of related proteins for which no known function has been found. The number of genes and their function for each group have been determined by combining results from clustering, using the ISODATA algorithm, with data from the literature and public databases. Primarily HGNC and UniProt were used. Consensus TM helix numbers have been set through evaluation of data from external resources among the literature and databases together with prediction results from Phobius, SOSUI, and TMHMM.

We created an overview of the occurrence of structures with a certain membrane topology, that is, the number of TM α-helices and the localization of the N-terminal, in the different functional classes based on predictions with Phobius (Figure 6). This clearly shows that 1 TM proteins are the most numerous structures and that the number generally decreases with increasing numbers of TM helices. However, the 7 TM structure is an outlier, having the second highest count. It is also possible to see a trend where some topologies are more common for certain classes. Receptors have in general a 1 TM or 7 TM topology representing 29% and 52% of all receptors respectively, whereas 71% of the transporters have more than 6 TM. It is also evident that the fraction of unclassified proteins is greater for low numbers of TM helices. 1 TM and 2 TM topologies contribute to 77% of the unclassified proteins.

### Availability and further analysis

The classification together with predictions by Phobius, SOSUI, and TMHMM for each protein is available in Additional file 1. Sequences in FASTA format for each class and a BLAST database for the whole dataset can be found in Additional file 2. To help readers in making their own analysis of the dataset a short user guide is provided as Additional file 3. For additional data or support with extended searches and analysis of the dataset and classification, feel free to contact the corresponding author.

## Discussion

We provide a non-redundant dataset for the human membrane proteome and a qualitative functional classification for all major groups and families containing 6,718 proteins. Comparison with the most recent and reliable set of the genes in the human genome [1] suggests that the 5,359 validated protein coding α-helical transmembrane proteins comprise 27% of the entire human proteome. The relative number of membrane proteins coded in the human genome was estimated to be 20% after the finishing of the human genome sequence [3]. The overall portion of membrane bound proteins has thus increased with 7 percentage points, whereas the estimated number of genes has decreased significantly from 31,778 [3] to 19,523 [1]. This may suggest that the identity of membrane proteins in general has been more reliable than soluble proteins, which may reflect that they receive relatively large attention in medical research because of their role as drug targets. Our number of 27% is within the two previously suggested spans of 15 to 39% [4] membrane proteins in the human proteome and 20 to 30% [2] in any proteome, regardless of species. It is notable that our clustering and classification resulted in 3,145 (59%) valid proteins of the total dataset being identified to belong to 234 families or groups with at least two members, while 41% of the data set are single genes with no clear identity (significantly lower than 13% (*P *< 0.001)) to any other human membrane protein coding gene. We are not aware of any exact estimate of the relative percentage of genes belonging to protein families within the human genome but one previous estimation has suggested that at least 40% of the human genes are members of gene families [15].

### Comparison of membrane proteome composition

The largest functional group of membrane proteins is the Receptor class, constituting 23% of our membrane proteome dataset and 40% of the classified proteins. This is in large contrast with the membrane proteome of *E. coli *where receptors count for only 5% of the membrane proteome, leaving transporters as the most prominent group with 40%, compared with 15% in humans and 32% in *S. cerevisiae*'s membrane proteomes [5,6]. The estimated number of membrane receptor proteins has increased from about 35 in *E. coli *to over 1,000 in humans. The GPCRs (7 TM) count for the largest expansion, with 67% of the human receptors compared with zero in bacteria and three proteins in *S. cerevisiae *[16]. The major expansion of the receptors within the metazoan lineage may reflect the need for a diverse repertoire of signaling systems for communication between cells in more complex, multicellular organisms [16-18].

### Membrane topology and protein function

**Figure 7:**
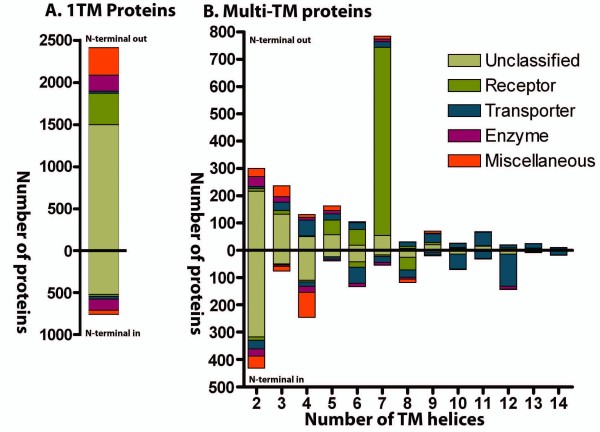
**Transmembrane topology analysis**. The graphs show the distribution of proteins with different membrane topologies for the membrane proteome. Proteins with the N-terminal located on the outside of the membrane are plotted upwards and those with the N-terminal on the inside are plotted downwards. The colors of the bars represent the proportion of different functional groups and unclassified proteins for a topology. The topologies have been predicted for each protein with Phobius. **A**. The graph shows the distributions of proteins predicted to have one TM helix. **B**. The graph shows the distribution of proteins predicted to have multiple TM helices. Forty-three proteins with more than 14 TM helices have been excluded from graph B.

Common for all membrane proteins in this study are that their amino acids form membrane crossing hydrophobic α-helices and their function depends on the number of helices and the orientation of the N- and C-terminal. Our results show a clear bias in favor of certain topologies within the membrane proteome. We find that the majority of the proteins with odd numbers of TM helices have their N-terminal positioned on the outside of the membrane, whereas the opposite is true for those with an even number (Figure 7). Hence, it is more common for proteins to have their C-terminal oriented to the inside of the membrane. We find that this is true for 75% of the TM proteins predicted by Phobius. Previously it has been reported that 82% of the TM proteomes of *S. cerevisiae *have the C-terminal located on the cells' inner side [6]. It is common that the C-terminal part of membrane proteins interacts with other intracellular proteins, for example, G-proteins and many accessory proteins bind to the C-terminal of GPCRs and this is essential for signaling [19]. Many proteins have only one single TM helix (47%) and while some of these TM regions seem to have a primary role to simply anchor the protein to the membrane, that is, no signal or substrate is relying on the TM helices to cross the membrane, several form oligomers that can participate in the signal process. Non-GPCR receptors are common 1 TM proteins (Phobius predicts 397 receptors to have this topology) and 60% of the proteins in the enzyme group are also found here, but only 57 transporters. On the other hand, multi-TM proteins are often highly dependent of the arrangement of their TM helices that form complex structures. Transporters are the most obvious group, which in general has high TM numbers; 77% of the transporters have at least a 6 TM topology and 76% of the classified proteins with at least 8 TM helices are transporters. This is also true for a majority of the families classified as transporters (Figure 4). In the TCDB database, which holds transporter families from all organisms, 70% of 2,847 α-helical channels, secondary transporters and active transporters, have at least a 6 TM topology according to predictions by Phobius. Further, 12 TM was the most common topology in TCDB (17%), which is in analogy with 16% in human (Figure 7 and Additional file 1). Thus, a high number of TM helices is a good predictor for transporter function and the topologies of the human transporter are representative for transporters in general, considering a number of distant species. There are also receptors with a high number of TM helices. The 7 TM structure of the large GPCR group, which contains 67% of the human receptors, undergoes a conformation change upon ligand binding, which propagates the signal into the cell. Other topologies among the receptors other than 1 TM and 7 TM are only found in seven of the 61 classified receptor groups, a total of 26 proteins. One of these are the two Hedgehog receptors, Patched, with a 12 TM topology which otherwise is almost exclusively found among transporters. In the 74 groups of the Miscellaneous class, some interesting observations regarding membrane topology are made (Figure 6). In the group of Structure/Adhesion proteins, with 187 members, all 12 families show either a 1 TM or 4 TM topology. Most numerous are the nine families of 1 TM proteins, 73% of the group, which among others contains the large cadherin and protocadherin families of 30 and 38 proteins, respectively. These proteins generally form homologous connections between cells, that is, two identical proteins from the cells adhere to each other [20]. They are important during development and for keeping tissue integrity and morphology, since cells expressing the same proteins stick together. In addition, there are also some minor 1 TM families, for example, Sarcoglycan, that are found in structures that anchor the muscle fibers to the extracellular matrix [21]. The remaining 27% of this group are 4 TM proteins of Structure/Adhesion character that can be divided into three families; gap junctions (connexins), claudins, and five proteins of the EMP/PMP22/LIM family.

The members of the two latter families belong to the same Pfam domain (PF00822) and are likely to share common descent. In the Pfam database we find that 58% of the 1,113 non-human proteins containing any of the Pfam families found in the Structure/Adhesion class, except immunoglobulins, are predicted to possess only one TM while 20% are predicted to have a 4 TM topology. This suggests that the relative frequency of 1 TM and 4 TM Structure/Adhesion proteins is similar in humans and other organisms. Proteins with a 4 TM structure are also found in the 'Other' and 'Protein of unknown function' groups, constituting 25% and 16% of the respective groups. Hence, the 4 TM proteins are over-represented in the Miscellaneous class, but they are also found to a rather high extent among transporters, especially in the families of ion channels. It should be noted that the 4 TM proteins of transporters and the Miscellaneous class generally are predicted to have opposite topology: the transporters have their N-termini located on the outside of the membrane whereas the Miscellaneous proteins have it on the inside. The 4 TM topology proteins which are found in the Miscellaneous class are not classical receptors or enzymes, but rather involved in the formation of structures (for example, claudin), vesicle trafficking (for example, synaptogyrin and synaptophysin) or organizing other proteins of the membrane (for example, tetraspanins) [22-24]. Therefore they could have been ignored in pharmacological research efforts, which explain why many of the 4 TM proteins are found in this group. In general, it seems that 4 TM topology proteins are relatively under-studied. Overall it is evident that a larger portion of the short proteins with fewer than seven helices, and especially 1 TM proteins, are uncharacterized. Such bias has been shown for other organisms in previous studies [5,6].

### Identification of uncharacterized proteins and families

Our clustering resulted in the identification of new protein groups and novel members of existing families. Discussions about individual members in existing families are found in Additional file 4. Here we want to highlight five clusters with a total of 41 sequences found in the Miscellaneous class where no previous relationship within the families have been reported. These families are simply termed New TM Group (NTMG): NTM1G1, NTM1G2, NTMG1, NTMG2, and NTM5G1; they are more or less uncharacterized with little annotation and no similarity to any Pfam domain. The NTM5G1 family contains three proteins; two are found in a cluster at chromosome 11 and one at chromosome 4. Two are predicted to be 5 TM proteins and one 3 TM. They show high identity to the C-terminal end of the 11 TM protein *Unc93B1*. This protein is found in chromosome 11, implicating an expansion of the family through a gene duplication event. Recently *Unc93B1 *was reported to be involved in trafficking of toll receptors to endolysosomes and is proposed to be involved in immunodeficiency, but when its homolog was initially characterized in *C. elegans *it was found to be involved in muscle contraction, which suggests multiple functions for the putative family [25,26]. The three novel proteins have orthologs in several species, which supports them as valid proteins and they might represent a novel subfamily of truncated *Unc93B1 *homologues (data not shown). Such truncated genes were discussed by Kashuba and colleagues as they found clones with high similarity to the 3' part of the *Unc93B1 *gene [27]. None of the other NTMG families has any similarities with known proteins and can thus be considered as virtually uncharacterized. Considering the extent of our clustering methods we find it unlikely that larger groups of closely related proteins are left to be discovered within the human TM proteome, although it cannot be entirely excluded. However, there are probably still several distant members of existing families and diverse novel families that could be identified in the future using more sensitive techniques than sequence comparisons.

## Conclusion

We annotated the majority of the human membrane bound proteome linked with functional properties and family or group affiliation. This classification represents most major families and groups found in the membrane proteome and provides the most detailed and updated count of their members. Overall, our clustering and functional classification approach is likely to be useful in order to create a detailed map of the entire human genome.

## Methods

### Retrieval of the initial membrane proteome dataset

IPI Human version 3.39 was downloaded from EBI containing 69,731 protein sequences [11]. Membrane topology was predicted for the IPI Human dataset by the use of three different applications to improve the accuracy [4]: Phobius, TMHMM, and SOSUI [2,12,13]. Phobius and TMHMM both use hidden Markov models (HMM) to predict membrane topology, but different training sets have been used and Phobius also uses a HMM to predict signal peptides. SOSUI evaluates amino acid hydrophobicity and amphiphilicity for its predictions and complements the HMM methods as it is not dependent on training sets. These three programs predict the topology of TM proteins spanning the membrane with α-helices. Thus, we consider only such membrane proteins in this study.

First Phobius was used to predict TM helices and signal peptides for the IPI Human dataset. The predicted signal peptides were cut out of the sequences before prediction with SOSUI and TMHMM to avoid false-positive prediction as suggested by Ahram and colleagues [4]. Candidate membrane proteins were initially selected as those predicted to have TM helices by at least two applications. The TM predictions received from the three individual applications and after the consensus approach can be found in Figure 1. The number of protein sequences was reduced by aligning all protein sequences to the human genome with BLAT and selecting the longest sequence from each group of overlapping sequences [14]. Consequently, only one representative protein sequence for each genomic locus was kept and alternative splice variants and so on were discarded. This non-redundant membrane proteome dataset was used in the clustering.

### Clustering

The clustering was performed with a local implementation of the ISODATA algorithm [28] with improvements according to Philips [29]. The algorithm initially chooses a number of random data as cluster centroids and assigns all the remaining data to the closest cluster centroid. The clusters are evaluated; clusters with fewer than three members are dropped and clusters where the standard deviation of the internal distances is above an empirically determined threshold are split. Finally, new centroids are calculated as the average of the data in each cluster, respectively. This procedure is iterated for a selected number of times or until the clusters do not change between two iterations. In our implementation, each protein was represented by a vector containing distances to every other protein in the dataset. Distances were calculated as the score from a Needlemann-Wunsch global alignment [30] normalized by the alignment length as produced by the implementation in the EMBOSS package run with standard parameters [31]. In the clustering, Euclidean distance was used between data. The method was stabilized by making several runs. Consensus clusters were created by letting the data that shared clusters in all runs define kernels. Each datum was assigned to the kernel which it clustered with the most times and clusters were merged if a datum was associated to several clusters in more than 50% of the runs. BLAST [32] and HMMER [33] was used to confirm and mine each accepted cluster. HMMs for each cluster were constructed by iteratively constructing multiple alignments with Kalign [34] and removing columns represented in less than 10% of the sequences, or in at least two sequences if the cluster contains less than 10 sequences. A tagged BLAST database was constructed from the clusters and a background consisting of the proteins for which no TM helices were predicted. The background set was reduced during the procedure, as non-TM members of families were discovered, for example, kinases [35]. Proteins which had at least four of the top five unique BLAST hits with *E*-values below 0.01 and matching the best hits cluster-HMM were automatically assigned to that cluster. Exceptions were made if the query cluster contained less than five transcripts; then it was enough to hit all query transcripts in order for a protein to be assigned to that cluster. The whole cluster procedure was repeated seven times on the remaining unclustered proteins after each iteration. The created clusters were used as a starting point for the classification.

### Classification

The sequences of the clusters were assigned with GO terms [36], describing molecular function, and Pfam [37] families retrieved from the Gene Ontology website and the IPI human annotations. This information, together with the IPI annotation, was used to receive an initial view of the clusters' protein family affiliations and the general function of the families. The clusters were sorted into one of four classes depending on their type function: receptors, transporters, enzymes, or miscellaneous. The clusters with proteins that fitted into more than one class, such as ligand-gated ion channels (that is, transporter and receptor function) and receptor-type kinases (that is, enzyme and receptor function), were chosen and sorted according to one function. This was done by evaluating our data and literature to make the best and most objective choice possible. During the manual classification the clusters were compared in terms of members and function to external references in two steps.

### Step 1: comparison with group databases

During the first step we used general group databases specialized in receptors (HPMR) [18], transporters (TCDB) [38] or enzymes (BRENDA) [39]. The databases were examined with slightly different approaches (see below), due to differences in content and availability.

#### Receptors: the HPMR database

The HPMR database is accessible through its web interface and no sequence dataset could be downloaded. Thus, the comparisons between families assigned among the clusters and HPMR were performed by purely manual inspection. The HPMR website was examined and the different families of receptors were identified and compared with the cluster dataset, allowing clusters to be classified as the correct receptor family. Families found to be missing among the clusters were manually added by gathering sequences from the IPI dataset.

#### Transporters: the TCDB database

The TCDB database provides a hierarchical classification system, annotation, and information for each class of different transporters. A dataset with representative protein sequences for each transporter class is provided. However, the sequences are from various organisms and human sequences are not available for all transporters.

Multiple sequence alignments for each cluster were created following the same method as described for the clustering. The multiple sequence alignments were used to build and calibrate HMMs with HMMER, using standard parameters. The TCDB dataset was searched against the cluster HMMs with HMMER. A cluster was assigned the same transporter class as a sequence if the *E*-value was below 10^-6 ^and the HMM was the best hit for the sequence. In addition, TCDB were manually investigated through the website and families found to be missing in the clustering were gathered from the IPI dataset.

#### Enzymes: the BRENDA database

The BRENDA human dataset was downloaded from the website and aligned against the human genome using BLAT. Splice variants were removed and the longest representative for each genomic location was kept, using the same approach as described in the retrieval of the membrane proteome dataset. The BLAT results for the BRENDA dataset were checked for overlap with the cluster dataset. If two sequences overlapped they were considered to be representatives for the same gene and the cluster sequence was annotated as an enzyme of the same class as the BRENDA sequence. Clusters were then annotated with the same enzyme class as their containing sequences.

### Step 2: comparison with family specific resources

In the second step, comparisons were made between the clusters and resources containing family oriented data such as records of family members and information about family function and structure. The resources were both general (for example, HGNC [40] and UniProt [41]) and family specific (for example, web-resources such as KinBase [35] and SLC-tables [42] or the literature). This was performed by manual inspection and/or bioinformatic approaches, similar to Step 1. The clusters of the miscellaneous families were carefully examined and annotated after comparison with HGNC and UniProt. The group was divided into four groups: Structure/Adhesion, Ligand, Other, and Proteins of unknown function. The two latter groups contain protein families with functions that did not fit in the other groups and protein families of unknown function. The manual curation allowed for sequences and families that were missing in the clusters to be added. This made it possible to construct as complete a record as possible for the major protein families and classify it according to functionality. If possible, the number of consensus TM helices was set for each family. This was performed by manual evaluation of the prediction results from TMHMM, SOSUI, and Phobius together with data from the literature.

### Estimating the size of the membrane proteome

The IPI dataset has the ambition to contain all known protein sequences, not only the well established. Thus, the use of IPI in the analysis limits the possibility to miss less characterized proteins for the cost of more false positives, such as protein sequences from gene prediction artifacts and non-coding genes that occurs in this dataset. This complicates the estimation of the number of membrane proteins. To address this issue we have used a dataset created by Clamp and colleagues based on the union of the human gene catalog of Ensembl 35 and 48 where all genes have been classified as protein-coding or non-coding [1,43]. All transcript sequences representing the genes in this dataset were downloaded from the Ensembl FTP-site [44] and aligned against the human genome with BLAT. The transcripts were checked for exon overlap with our membrane dataset. If a membrane dataset sequence overlapped a Clamp-sequence it inherited its class. Consequently, we can make a more critical estimation of the number of genes coding for membrane proteins.

## Abbreviations

HMM: hidden Markov model; IPI: International Protein Index; NTMG: New TM Group; TM: transmembrane.

## Authors' contributions

MSA carried out the study, analyzed the data, participated in the design, and drafted the manuscript. KJVN implemented the cluster algorithm and participated in the design of the study and in the writing of the manuscript. RF and HS conceived the study, and participated in its design and the writing of the manuscript. All authors read and approved the final manuscript.

## Supplementary Material

Additional file 1 **The table contains the International Protein Index (IPI) accession numbers and classification for the final membrane protein dataset together with predictions by Phobius, SOSUI, and TMHMM.** Click here for file

Additional file 2 **ZIP-archive containing FASTA files for all classes and a BLAST database for the entire dataset.** Click here for file

Additional file 3 **A short user guide for searching and further analysis of the dataset and classification.** Click here for file

Additional file 4 **This text describes putative novel protein families and members of existing families.** Click here for file
