# Supplementary material for: Mapping the human membrane proteome: a majority of the human membrane proteins can be classified according to function and evolutionary origin
Source: BMC Biol. 2009 Aug 13;7:50. doi: 10.1186/1741-7007-7-50 (PMC2739160; doi:10.1186/1741-7007-7-50)
Supplement: Additional file 3 — A short user guide for searching and further analysis of the dataset and classification. [file 1741-7007-7-50-S3.doc]

# User guide: Handling and Searching the Human Membrane Protein Dataset

This short guide will try to aid users of our dataset and classification of the human membrane proteome in making searches and investigations of their own. It primarily addresses users with no or little experience in handling larger tables and the popular sequence alignment tool BLAST.

1. **The classification and prediction table – Additional Datafile 1**

This Excel table contains IPI accession numbers and classification for the final dataset together with the final transmembrane predictions by Phobius, SOSUI and TMHMM. The Excel table can be opened and handled in OpenOffice.org Calc, but of course also Microsoft Office Excel. All instructions are for OpenOffice.org (OO) 3.0, as it is freely available for several operating systems, but they should apply for Excel with only small adjustments.

*A basic search*

A good search function is available in OO by pressing the *Ctrl + F* buttons. All information in the table is searchable by filling in the search field and then click on the search button.

To search for a gene of special interest*,* primarily use its standard gene symbol, e.g. *KCNE1,* to retrieve information about classification, predictions and its IPI accession number. The accession number can be used to search the IPI database <http://www.ebi.ac.uk/IPI/>where additional annotation and links to other resources is found.

*Filtering*

We also would like to encourage user’s to try the filter function (Menu*: Data => Filter => Standard Filter*) to customize the table view, e.g. all but one class could be filtered away or only show proteins with more than seven transmembrane helices predicted by Phobius.

*Pivot table*

OO’s Data table function is excellent for making customized views and fast analysis of data. It is available through *Menu: Data=>Data table => Start,* choose current selection. Create your Data table by drawing different boxes to the fields. For example:

- Place *Main Class* in *Row Fields* and *IPI Accessions* in *Data Fields*.
- Double click the *IPI Accession* box, choose count in the list and click OK.
- Click the *More* button and set *Results to new sheet* in the menu.
- Click OK.

This should create a Data Table showing the number of Proteins (by counting IPI Accession) for each Main Class (Receptor, Transporter, Enzyme, Miscellaneous and Unclassified). The Data table is a powerful tool which allows you to make fast and useful investigations of the dataset.

1. **Searching the Human Membrane Proteome for Homologous Sequences.**

Many users are probably interested in finding out what human protein and class a specific protein sequence is most similar to (homologous). This could for example be a protein sequence from a different species. The most popular application for this type of analysis is BLAST which can be downloaded from the NCBI website: (<http://blast.ncbi.nlm.nih.gov/Blast.cgi?CMD=Web&PAGE_TYPE=BlastDocs&DOC_TYPE=Download>)

Download and install blast for your platform according to the instructions (Windows, Linux etc.), it is required for performing the following analysis. The example below is described for Linux, but should be analogous to other operative systems, e.g. Windows.

Example: We are interested in finding which human membrane protein a particular *Arabidopsis thaliana* sequence is most similar and what class it might belong to. We have the following sequence in FASTA format and save it in the file MySequence.fa:

>Q9C8G9

MGFEPLDWYCKPVPNGVWTKTVDYAFGAYTPCAIDSFVLGISHLVLLILCLYRLWLITKD

HKVDKFCLRSKWFSYFLALLAAYATAEPLFRLVMRISVLDLDGAGFPPYEAFMLVLEAFA

WGSALVMTVVETKTYIHELRWYVRFAVIYALVGDMVLLNLVLSVKEYYGSFKLYLYISEV

AVQVAFGTLLFVYFPNLDPYPGYTPVGTENSEDYEYEELPGGENICPERHANLFDSIFFS

WLNPLMTLGSKRPLTEKDVWHLDTWDKTETLMRSFQKSWDKELEKPKPWLLRALNNSLGG

RFWWGGFWKIGNDCSQFVGPLLLNELLKSMQLNEPAWIGYIYAISIFVGVVLGVLCEAQY

FQNVMRVGYRLRSALIAAVFRKSLRLTNEGRKKFQTGKITNLMTTDAESLQQICQSLHTM

WSAPFRIIVALVLLYQQLGVASIIGALFLVLMFPIQTVIISKTQKLTKEGLQRTDKRIGL

MNEVLAAMDTVKCYAWENSFQSKVQTVRDDELSWFRKAQLLSAFNMFILNSIPVLVTVVS

FGVFSLLGGDLTPARAFTSLSLFSVLRFPLFMLPNIITQMVNANVSLNRLEEVLSTEERV

LLPNPPIEPGQPAISIRNGYFSWDSKADRPTLSNINLDIPLGSLVAVVGSTGEGKTSLIS

AMLGELPARSDATVTLRGSVAYVPQVSWIFNATVRDNILFGAPFDQEKYERVIDVTALQH

DLELLPGGDLTEIGERGVNISGGQKQRVSMARAVYSNSDVCILDDPLSALDAHVGQQVFE

KCIKRELGQTTRVLVTNQLHFLSQVDKILLVHEGTVKEEGTYEELCHSGPLFQRLMENAG

KVEDYSEENGEAEVDQTSVKPVENGNANNLQKDGIETKNSKEGNSVLVKREERETGVVSW

KVLERYQNALGGAWVVMMLVICYVLTQVFRVSSSTWLSEWTDSGTPKTHGPLFYNIVYAL

LSFGQVSVTLINSYWLIMSSLYAAKKMHDAMLGSILRAPMVFFQTNPLGRIINRFAKDMG

DIDRTVAVFVNMFMGSIAQLLSTVILIGIVSTLSLWAIMPLLVVFYGAYLYYQNTSREIK

RMDSTTRSPVYAQFGEALNGLSSIRAYKAYDRMAEINGRSMDNNIRFTLVNMAANRWLGI

RLEVLGGLMVWLTASLAVMQNGKAANQQAYASTMGLLLSYALSITSSLTAVLRLASLAEN

SLNSVERVGNYIEIPSEAPLVIENNRPPPGWPSSGSIKFEDVVLRYRPELPPVLHGVSFL

ISPMDKVGIVGRTGAGKSSLLNALFRIVELEKGRILIDECDIGRFGLMDLRKVLGIIPQA

PVLFSGTVRFNLDPFSEHNDADLWESLERAHLKDTIRRNPLGLDAEVTEAGENFSVGQRQ

LLSLARALLRRSKILVLDEATAAVDVRTDVLIQKTIREEFKSCTMLIIAHRLNTIIDCDK

VLVLDSGKVQEFSSPENLLSNGESSFSKMVQSTGTANAEYLRSITLENKRTREANGDDSQ

PLEGQRKWQASSRWAAAAQFALAVSLTSSHNDLQSLEIEDDNSILKKTKDAVVTLRSVLE

GKHDKEIEDSLNQSDISRERWWPSLYKMVEGLAVMSRLARNRMQHPDYNLEGKSFDWDNV

EM

In Additional Datafile 3 a BLAST database for the human membrane proteome is available in the folder BlastDB (created with *formatdb -i BlastDB.fa -o F*).

- Open a terminal window (command prompt).
- The *blastall* program from the BLAST software package is used. Change *path* to the location of the files on your computer. Type the following command:

*blastall -p blastp –d path/BlastDB/BlastDB.fa -i /path/MySequence.fa -o /path/MyResults.txt*

(More information about blastall can be retrieved by typing *blastall --help)*

The results of our BLAST search are found in MyResults.txt. The file contains the following list with the best hit first (only showing top three hits):

Score E

Sequences producing significant alignments: (bits) Value

IPI00023868 879 0.0

IPI00719178 852 0.0

IPI00006674 800 0.0

An E-value close to zero indicates a homologous hit (should be <10^-5^). When searching for IPI00023868 in the table in Additional Datafile 1, using OpenOffice.org Calc, we find that it is an ABC transporter (*ABCC2)*. Thus, it is likely that our *A. thaliana* protein belongs to this class, which is correct according to UniProt.

**Conclusion**

By using standard tools such as OpenOffice.org it is possible to make powerful analysis of the data and classification and by including quite simple blast searches it could be extended to cover other species. We would like to encourage users to contact the authors for support with more advanced analysis or questions about the dataset our classification
