# Supplementary material for: Mapping the human membrane proteome: a majority of the human membrane proteins can be classified according to function and evolutionary origin
Source: BMC Biol. 2009 Aug 13;7:50. doi: 10.1186/1741-7007-7-50 (PMC2739160; doi:10.1186/1741-7007-7-50)
Supplement: Additional file 4 — This text describes putative novel protein families and members of existing families. [file 1741-7007-7-50-S4.doc]

**Supplementary information S2:**

**New groups of transmembrane proteins:**

Here we want to highlight clusters of five previously unstudied families with a total of 41 sequences found in the Miscellaneous class. These families are simply termed New TM Group (NTMG); NTM1G1, NTM1G2, NTMG1, NTMG2 and NTM5G1. None of these novel families contains proteins with known Pfam domains and they lacked detailed annotation. The NTM5G1 family contains three proteins, two are found in a cluster at chromosome 11 and one at chromosome 4. Two are predicted to be 5TM proteins and one 3TM. They show high identity to the C-terminal end of the 11TM protein Unc93B1. This protein is found in chromosome 11, implicating an expansion of the family through a gene duplication event. Recently Unc93B1 was reported to be involved in trafficking of toll receptors to endolysosomes and is proposed to be involved in immunodeficiency, but when its homolog was initially characterized in Caenorhabditis elegans it was found to be involved in muscle contraction, which suggests multiple functions for the putative family (1, 2). The three novel proteins have orthologs in several species, which supports them as valid proteins and they might represent a novel subfamily of truncated Unc93B1 homologues (data not shown). Such truncated genes were discussed by Kashuba and colleagues as they found clones with high similarity to the 3’ part of the Unc93B1 gene (3). None of the other NTMG families have any similarities with known proteins and can thus be considered as virtually uncharacterized.

Putative solute carriers:

We have identified two novel protein families as putative members of the Drug metabolite transporter (DMT) group; the NIPA family and the AMAC family. Previously, the SLC35 family of nucleoside-sugar transporters has been the only reported human family of the group (4). Both of the novel families contain six proteins each with a 9TM structure. Members of the NIPA family are known to be involved in neurodegenerative disorders, such as hereditary spastic paraplegia, and have been shown to transport Mg^2+^ (5). The proteins are predicted to contain the Pfam domain DUF803 (PF05653), which belongs to the DMT clan. AMAC is an abbreviation of Acyl-malonyl condensing enzyme. This name and the enzymatic function were proposed by Kells and Maynard based on what appeared to be sequence similarities between the *AMAC1* gene from mouse and the *Arabidopsis thaliana* (AT) *FAE1* gene, an enzyme catalyzing the elongation of fatty-acids (6). The results were based on comparisons of partial sequences retrieved from RT-PCR experiments. However, when we compare the whole sequence of mouse and human *AMAC1* to AT’s *FAE1* no significant homology is found, which suggests that the former annotation is incorrect. Instead the AMAC proteins are found to contain the Pfam domain DUF6 (PF00892), which is a member of the DMT clan. The number of TM helices predicted for the proteins of these two families and the affiliation to the DMT clan suggests that they belong to the SLC superfamily.

In addition to the two putative SLC families, we have identified 15 putative novel members in nine of the existing 46 families. One of these putative SLCs, *TMEM104*, is clustered with the SLC32, SLC36 and SLC38 families. These are families of amino acid transporters belonging to the APC group and are among other important for the transportation of amino acids in the cells of the CNS (7). We have previously reported these families’ close relationship and expected common origin, which is supported by the families’ formation of a single mutual cluster in this study (8). *TMEM104* are poorly characterized, but contains a Pfam domain that is common among the SLC38 and SLC36 family members (PF01490) and have 10TM which are in line with the APC family. Thus, *TMEM104* have strong support for being a valid protein of the SLC32, SLC36 and SLC38 families.

**A putative novel** **Calcium channel gamma subunit**

The ten calcium-channel γ-subunits form the largest auxiliary transport protein family, i.e. proteins that modulate transport rather than perform it. They are 4TM proteins with structural similarities to the Claudin and the EMP-PMP22-LIM families (9) At first the family was thought to interact with the α-subunit of the voltage-gated calcium channels, inhibiting calcium currents. However, this has only been proven for two members, whereas a function as trafficking regulators of AMPA receptors of the glutamate cationic channels family have been established for four of the proteins, called TARPS. Thus, this is a functionally diverse family and there are still several uncharacterized members. The clustering process has uncovered one putative novel member (IPI00002685) of this family which clustered with uncharacterized proteins and the four TARPS, but not with the two calcium current modulators. However, the family characteristic GLW motif of the first extracellular loop is present in our candidate protein, encouraging the addition of the sequence to the family.

A putative novel G-protein coupled receptor:

The clustering revealed a novel protein related to the two GPCRs GPR172A and B called C20orf54. These two proteins are predicted to have a 10TM structure and contain the Pfam domain DUF1011, which are in concert with the predictions for the novel protein. The GPR172 proteins are largely unexplored atypical members of the GPCR superfamily. However, Ericsson et al. reported them to be receptors for pig endogenous retroviruses, which are of importance for xenotransplantation, and more recently Andriamampandry and co-workers suggested that GPR172A is a receptor for gamma-hydroxybutyrate (GHB) (10, 11). This is a substance that both occurs naturally in the central nervous system and has been used as an anesthetic drug. Conclusively, this is an interesting family of potential therapeutic importance in the future and the novel member clearly illustrates the many remaining discoveries within the GPCR superfamily.

References

1. Kim YM, Brinkmann MM, Paquet ME, & Ploegh HL (2008) UNC93B1 delivers nucleotide-sensing toll-like receptors to endolysosomes. (Translated from eng) *Nature* 452(7184):234-238 (in eng).

2. Levin JZ & Horvitz HR (1992) The Caenorhabditis elegans unc-93 gene encodes a putative transmembrane protein that regulates muscle contraction. (Translated from eng) *The Journal of cell biology* 117(1):143-155 (in eng).

3. Kashuba VI*, et al.* (2002) hUNC93B1: a novel human gene representing a new gene family and encoding an unc-93-like protein. (Translated from eng) *Gene* 283(1-2):209-217 (in eng).

4. Ishida N & Kawakita M (2004) Molecular physiology and pathology of the nucleotide sugar transporter family (SLC35). (Translated from eng) *Pflugers Arch* 447(5):768-775 (in eng).

5. Goytain A, Hines RM, El-Husseini A, & Quamme GA (2007) NIPA1(SPG6), the basis for autosomal dominant form of hereditary spastic paraplegia, encodes a functional Mg2+ transporter. (Translated from eng) *The Journal of biological chemistry* 282(11):8060-8068 (in eng).

6. Kells AP & Maynard PV (1997) RT-PCR of fatty acid elongases. (Translated from eng) *Biochemical Society transactions* 25(1):20S (in eng).

7. Fredriksson R, Nordstrom KJ, Stephansson O, Hagglund MG, & Schioth HB (2008) The solute carrier (SLC) complement of the human genome: phylogenetic classification reveals four major families. (Translated from eng) *FEBS letters* 582(27):3811-3816 (in eng).

8. Sundberg BE*, et al.* (2008) The evolutionary history and tissue mapping of amino acid transporters belonging to solute carrier families SLC32, SLC36, and SLC38. (Translated from eng) *J Mol Neurosci* 35(2):179-193 (in eng).

9. Chen RS, Deng TC, Garcia T, Sellers ZM, & Best PM (2007) Calcium channel gamma subunits: a functionally diverse protein family. (Translated from eng) *Cell biochemistry and biophysics* 47(2):178-186 (in eng).

10. Ericsson TA*, et al.* (2003) Identification of receptors for pig endogenous retrovirus. (Translated from eng) *Proceedings of the National Academy of Sciences of the United States of America* 100(11):6759-6764 (in eng).

11. Andriamampandry C*, et al.* (2007) Cloning and functional characterization of a gamma-hydroxybutyrate receptor identified in the human brain. (Translated from eng) *Faseb J* 21(3):885-895 (in eng).
